# Virome diversity and molecular characterization of two emerging RNA viruses in mosquito populations from Yantai, China

**DOI:** 10.1128/msphere.00539-25

**Published:** 2025-12-09

**Authors:** Meixi Ren, Yumei Liu, Yongqin Wang, Yingxin Tu, Yaqing Guo, Xiaodong Sun, Guoyu Niu, Yanyan Wang

**Affiliations:** 1 Shandong Second Medical University 372527 , Weifang, China; 2 Weifang People's Hospital, Shandong Second Medical University 372527 , Weifang, China; 3 Suqian First Hospital 710134 , Suqian, China; University of California Davis, Davis, California, USA

**Keywords:** mosquito-borne viruses, *Culex quinquefasciatus*, novel viruses, phylogenetic analysis introduction

## Abstract

Mosquito-borne viruses represent a major global public health threat, with transmission dynamics governed by climatic, ecological, and anthropogenic factors. Yantai City, Shandong Province, situated in a warm-temperate monsoon climate zone, shares geographical and ecological characteristics with regions where mosquito-borne viruses are endemic, creating potential for virus introduction. We used metagenomics to systematically analyze viral communities in mosquitoes from the Yantai region. We collected 8,111 mosquitoes representing four genera and six species, with *Culex* being predominant (89.8%). High-throughput sequencing revealed 11 viral species spanning 9 families, including Peribunyaviridae and Picornaviridae. Notably, Serbia mononega-like virus 1 and Biggievirus Mos11 represent the first reports from China, with quantitative reverse transcription PCR revealing minimum infection rates of 0.34% and 0.68%, respectively. Phylogenetic analysis revealed close relationships to known viral strains, with several isolates potentially representing novel genera or species. Analysis revealed that *Culex quinquefasciatus* harbored the greatest viral diversity (five species), with significantly higher viral diversity in agricultural versus urban areas (*P* < 0.001). Several viruses demonstrated cross-species transmission potential, including Zhee mosquito virus, Zhejiang mosquito virus 3, and *Culex tritaeniorhynchus* rhabdovirus, all detected across multiple mosquito species. While most viruses appear mosquito-specific, several show close phylogenetic relationships to known pathogens, potentially posing public health risks warranting surveillance. This study addresses knowledge gaps regarding mosquito-borne viruses in the Bohai Rim region and provides a scientific foundation for regional viral surveillance and early warning systems.

**IMPORTANCE:**

Mosquito-borne viruses are a significant global health threat, with the potential to cause widespread disease outbreaks. This study investigated the viral diversity within mosquito populations in Yantai, China, and characterized the molecular features of two emerging RNA viruses. These findings highlight the remarkable viral diversity harbored by Culex mosquitoes and reveal higher viral diversity in agricultural areas compared to urban settings. Several identified viruses exhibit cross-species transmission potential and close phylogenetic relationships to known pathogens, suggesting that they may pose public health risks. Understanding these interactions is essential for predicting how environmental changes may affect virus transmission and the resilience of surveillance and control strategies.

## INTRODUCTION

Mosquito-borne viruses constitute important pathogens transmitted primarily through mosquito bites that infect vertebrate hosts and cause disease. Mosquitoes serve as major arthropod vectors, harboring and transmitting diverse viruses that comprise over half of all known vector-borne pathogens. Hundreds of mosquito-borne viruses have been identified globally, with approximately 100 demonstrating pathogenicity and posing significant threats to human and animal health. Mosquito species exhibit distinct vectorial capacities based on their ecological niches: *Culex* spp. serve as primary vectors for Japanese encephalitis virus (JEV) and West Nile virus (WNV); *Aedes* spp. primarily transmit dengue virus (DENV), Zika virus (ZIKV), chikungunya virus (CHIKV), and yellow fever virus, whereas *Anopheles* spp. serve as vectors for malarial parasites. Mosquito-borne viruses represent a major global public health threat, with transmission dynamics governed by climatic, ecological, and anthropogenic factors, including environmental factors such as temperature and rainfall (1–5).

Yantai City, Shandong Province (119°34′–121°57′E, 36°16′–38°23′N), is situated in the northeastern Shandong Peninsula, bordered by the Yellow and Bohai seas. The region exhibits a warm temperate monsoon climate with pronounced maritime influences. This latitude (~37°N) coincides with several global regions endemic for mosquito-borne viruses. Mediterranean coastal regions (Italy, Greece) represent WNV endemic zones, where outbreak intensity correlates with warm, humid conditions and enhanced vector breeding capacity at elevated temperatures (6). The Midwestern United States (Illinois, Ohio; 37°N–42°N) constitutes a WNV high-risk region, with transmission cycles dependent on summer temperatures and wetland ecosystems (7). Southern Korea (34°N–38°N) represents a historical JEV endemic region, where *Culex tritaeniorhynchus* density correlates positively with monsoon precipitation (8). These regions share climatic conditions (mean annual temperature 12°C–15°C, hot humid summers with abundant precipitation) and ecological features (coastal wetlands, migratory flyways, dense port infrastructure) with Yantai, creating favorable conditions for cross-regional viral transmission. As a major Belt and Road Initiative port hub, Yantai’s intensive international shipping and trade activities increase the risk of viral introduction through infected travelers or transported mosquito vectors (9).

This study used metagenomics to systematically characterize viral communities in Yantai mosquitoes, identifying 11 viral species across nine families, including *Peribunyaviridae* and *Picornaviridae*, and constructing phylogenetic trees to elucidate their evolutionary relationships. These findings address critical knowledge gaps regarding mosquito-borne viruses in the Bohai Rim region and demonstrate the diverse viral threats present in Yantai, providing a scientific foundation for regional surveillance and early warning systems.

## MATERIALS AND METHODS

### Collection and processing of mosquito samples

This study was conducted in rural agricultural areas and urban zones of Laizhou City, Yantai, Shandong Province. Ten standardized monitoring stations were established across diverse habitats, including pig farms, dairy facilities, goat pastures, and urban green spaces. Sampling was conducted during the peak summer season, with collections performed nightly from dusk to dawn (18:00–06:00). Mosquitoes were collected using UV light traps (365 nm; Kungfu Xiaoshuai, China). Collected specimens were transported in chilled biosafety containers and stored at −20°C until processing. Specimens underwent initial morphological identification and were grouped by collection site (50 individuals per pool) with comprehensive database documentation. Taxonomic identification was confirmed through cytochrome oxidase subunit I (*COI*) barcoding (10).

### Nucleic acid extraction

Pooled specimens were homogenized in 1,000 µL of pre-chilled DMEM culture medium at 4°C. Homogenization was performed using a cryogenic tissue homogenizer (Tissuelyser, Germany) at 30 Hz for 2 × 4 min with 1-min intervals until complete tissue disruption. Following centrifugation (15,000 × *g*, 30 min, 4°C), supernatants were collected, and total RNA was extracted using a viral RNA purification kit (TIANamp, TianGen, China). Extracted RNA was used immediately for downstream applications or stored at −80°C until analysis.

### High-throughput sequencing library preparation

Equal volumes (5 µL) of RNA from each pooled sample were combined to generate 19 sequencing libraries. RNA samples with concentrations ≥10 ng/µL underwent ribosomal RNA depletion using the FastSelect ribosomal RNA removal system (Vazyme, China), while low-concentration samples proceeded directly to library preparation. cDNA libraries were prepared using the V8 version high-throughput transcriptomics sequencing library preparation kit (Vazyme, China) following the manufacturer’s protocol. Library quality was assessed using an Agilent 2100 Bioanalyzer and quantified by quantitative (qPCR). Qualified libraries were converted to DNA nanoballs, loaded onto flow cells, and sequenced using paired-end 150 bp reads on a DNBSEQ-T7 platform.

### Sequence assembly and alignment

Raw sequencing data underwent multidimensional quality control using CLC Genomics Workbench, including base quality distribution assessment and adapter contamination screening. Low-quality sequences (Q-value <20) and redundant adapter sequences were removed using dynamic trimming algorithms, with reads <100 bp filtered out. STAR alignment was used to remove host-derived ribosomal RNA and genomic DNA sequences. Remaining high-quality reads were assembled *de novo* using MEGAHIT with k-mer sizes ranging from 21 to 121 (step size 10). Resulting contigs were identified using dual alignment strategies: BLASTX against the viral protein database (v2025.05) and BLASTN searches against the non-redundant nucleotide database. The Reference Viral Database public version was used (data retrieved through May 2025). Positive identification criteria were E-value <1 × 10⁻⁵ and sequence coverage >70%. Unique sequence clusters with sequencing depth >50 × and <60% homology to known viruses were designated as novel virus candidates for phylogenetic analysis and experimental validation.

### Virus taxonomic identification and functional annotation

Viral classification followed the International Committee on Taxonomy of Viruses (ICTV) taxonomy standards (Release 2025). Species identification was performed using whole-genome nucleotide alignments (NCBI nt database) and *RdRp* protein alignments (NCBI nr database). Novel species were defined by <80% genome nucleotide identity or <90% RNA-dependent RNA polymerase (RdRp) amino acid identity (11). Viral nomenclature followed the format: geographic location +host + taxonomic assignment. Study isolates of previously characterized viruses were designated with suffixes “21W-ZC” or “21W-GJD” to indicate collection sites. Viral genomes were analyzed for open reading frames (ORFs) using NCBI ORF finder (minimum length 300 bp/100aa), and functional domains were predicted using the Conserved Domain Database. Viral protein functions were predicted by comparison with known viral protein databases.

### Phylogenetic analysis

Sequence alignments were performed using the ClustalW algorithm within MEGA v10.2.6, with a gap opening penalty of 15 and a gap extension penalty of 6.66. Two phylogenetic methods were selected based on data characteristics: For known viral sequences, phylogenetic trees were constructed using the neighbor-joining (NJ) method with the Kimura two-parameter model, and node support was assessed through 1,000 bootstrap replicates. For newly discovered viral sequences: maximum likelihood analysis was performed using IQ-TREE 2.0, with the ModelFinder module selecting the best substitution model (BIC criterion), and branch support calculated via 1,000 ultra-fast bootstrap replicates. All phylogenetic trees underwent topological optimization and esthetic adjustment using FigTree v1.4.4, with key nodes labeled when branch support was >70%.

### Detection of newly identified viral RNA in mosquitoes

A one-step quantitative reverse transcription (qRT-PCR) detection system using TaqMan probes was established for viral nucleic acid detection in mosquitoes. The reaction mixture (25 µL total) contained the following: 5 × buffer (5 µL), dNTP mixture (1 µL), enzyme mixture (1 µL), forward and reverse primers (0.5 µL each, 10 µM), probe (0.25 µL, 5 µM), and template RNA (5 µL). The thermal cycling program consisted of the following: reverse transcription at 50°C for 30 min, initial denaturation at 95°C for 15 min, followed by 35 cycles of denaturation at 94°C for 30 s, annealing at 55°C for 30 s, and extension at 72°C for 1 min, with final extension at 72°C for 10 min. Positive results were defined by Ct values ≤ 35 and typical S-shaped amplification curves. Each experimental batch included no-template controls and positive controls (*in vitro* transcribed RNA) for quality assurance. Although metagenomics and qRT-PCR are powerful techniques for identifying viral genetic material, it is important to note that the detection of viral RNA does not provide definitive evidence of the presence of infectious virus particles.

### Data analysis and accession numbers

Nonparametric statistical methods were used to analyze viral carriage characteristics. The Mann-Whitney U test (α = 0.05) was employed to compare differences in viral species numbers between *Culex and mosquito species other than Culex*, combined with 10,000-iteration permutation tests to assess individual-level viral load distributions. Simpson’s 1 − D index (1 − ∑pi², where pi is the relative abundance of the *i*th virus) was used to quantify viral diversity within host groups, and 10,000-iteration permutation tests were conducted to compare viral species differences between farming areas and urban areas at the individual level. Community structure analysis was performed using the vegan package for PERMANOVA tests (Bray-Curtis distance matrix, 999 permutations), and transmission networks were visualized using multidimensional chord diagrams constructed with the circlize package. Three-dimensional structures of viral proteins were predicted using AlphaFold, followed by functional domain annotation using PyMOL. Novel virus prevalence was expressed as minimum infection rate (minimum infection rate [MIR] = number of positive pools/total number of tests × 1,000‰), and intergroup differences were assessed using Fisher’s exact test (*P* < 0.05 considered significant).

## RESULTS

### Virome profiles of mosquitoes collected from Shandong

**Fig 1:**
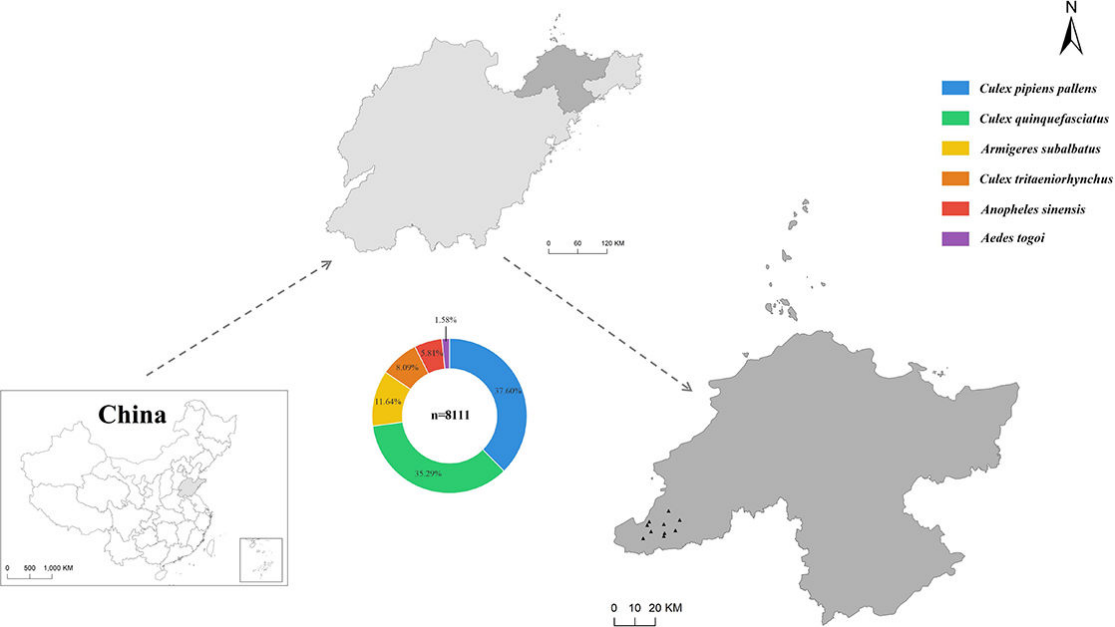
The map displays sampling locations and mosquito species composition in Shandong Province, China. Species are color-coded: blue (*Culex pipiens pallens*), green (*Culex quinquefasciatus*), orange (*Armigeres subalbatus*), yellow (*Culex tritaeniorhynchus*), red (*Anopheles sinensis*), and purple (*Aedes togoi*).

From June to August 2021, we collected 8,111 mosquitoes from 10 sampling sites in Yantai City, Shandong Province (Fig. 1). Morphological identification classified these mosquitoes into four genera and six species: *Culex pipiens pallens*, *Culex quinquefasciatus*, *Armigeres subalbatus*, *Culex tritaeniorhynchus*, *Anopheles sinensis*, and *Aedes togoi*. To confirm species identification accuracy, representative samples from each species were randomly selected for *COI* gene PCR amplification and sequencing, yielding fragments of approximately 603 bp. Sequencing results were compared with NCBI reference sequences using BLAST. Based on high homology (>98%) with reference sequences, mosquito species were ultimately confirmed. Among these, *Culex pipiens pallens* was most abundant (*n* = 3,050, 37.6%), while *Aedes togoi* was least abundant (*n* = 128, 1.6%). Mosquitoes were pooled into 165 tubes based on collection site and species, then processed for nucleic acid extraction and library construction, yielding 19 libraries (Table 1). High-throughput sequencing generated 143 Gb of data across the 19 libraries. After quality control and host sequence removal, 5,027,344,916 clean reads were obtained for downstream analysis, of which 53.9 million were viral reads (1.1% of total). *De novo* assembly yielded 12,804 viral contigs, which were classified into 11 virus species across nine families: *Picornaviridae*, *Peribunyaviridae*, *Tombusviridae*, *Narnaviridae*, *Botourmiaviridae*, *Solemoviridae*, *Xinmoviridae*, *Nodaviridae*, and *Rhabdoviridae*. None of the viruses were found in the host genome, excluding the likelihood of these viruses being present as endogenous viral elements.

**TABLE 1:** Primers and probes used for identification of the two newly identified viruses in this study

Virus	Type	Primer/probe	Sequence (5′to3′)
Serbia mononega-like virus 1	qRT-PCR	F	CGACACCACTGCTATGACAACTG
		R	TCATAAATAGGCGGATACTTGTCTTG
		P	CAACGTGGACATACCATTCTGTCTGCACC
Biggievirus Mos11	qRT-PCR	F	CCTTATTTTATAGTCTTCCTACGGATGTATT
		R	GTAATCCGTTCATAGTTGTAGATGTTTGT
		P	CTCAGACAGTGTCCGCTACTCCGATGATT

### Diversity, genome, and phylogenetic analysis of mosquito viromes

**Fig 2:**
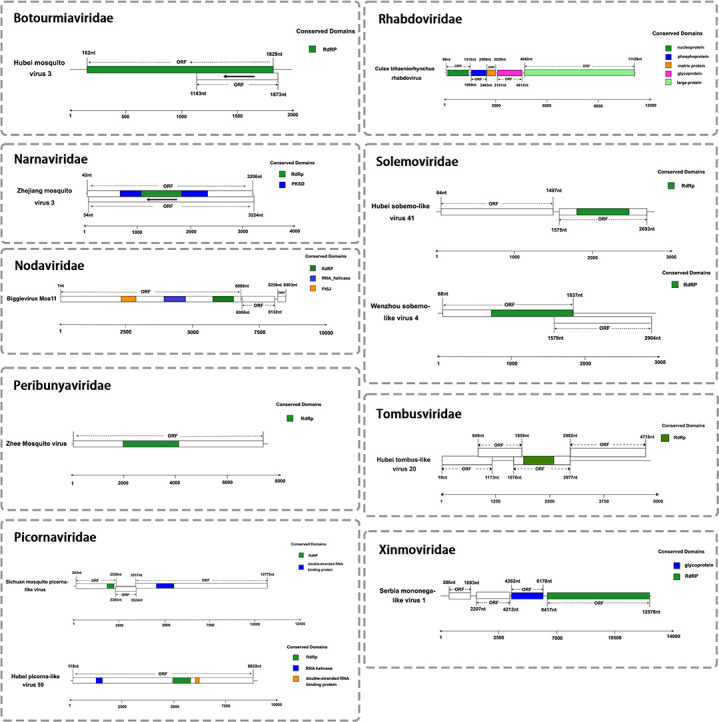
Genomic structures of 11 mosquito-associated viruses from nine distinct families identified by metagenomic sequencing in Shandong Province, China.

**Fig 3:**
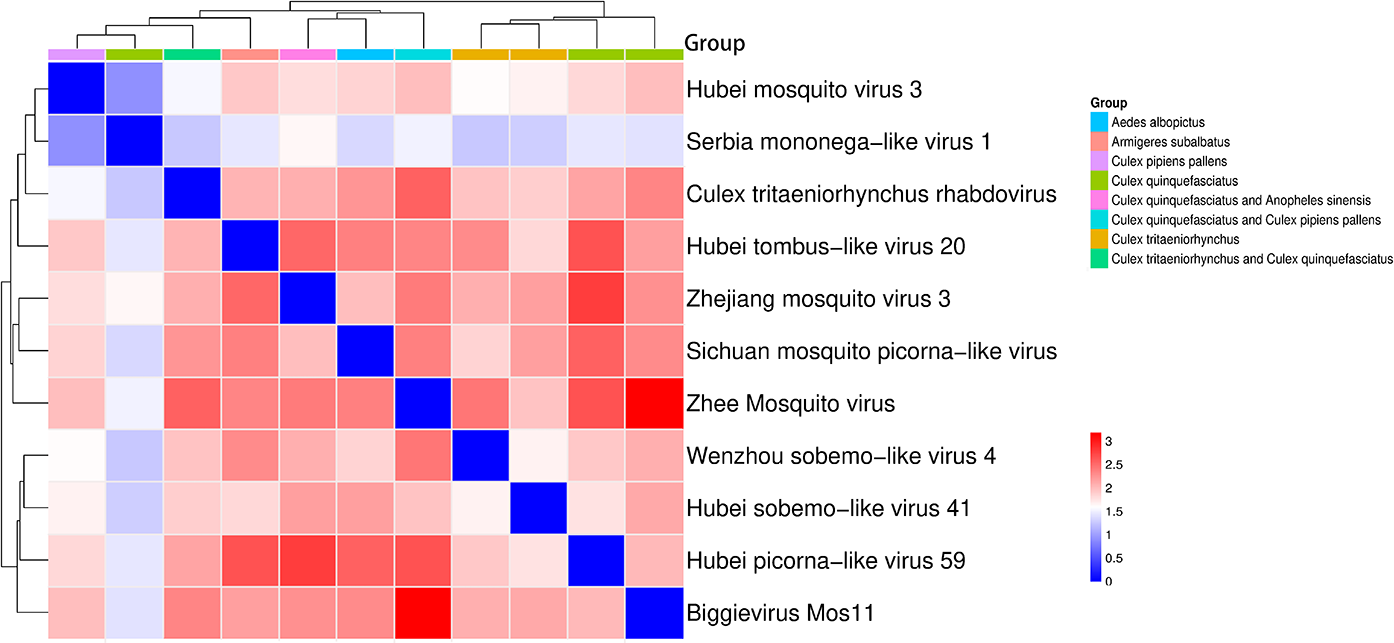
Heatmap analysis of viral sequence distribution across mosquito populations. Hierarchical clustering (left) shows evolutionary relationships among viral sequences (bootstrap values shown at nodes), while top clustering groups mosquito samples by similarity. Color bars indicate mosquito species. The gradient (blue to red) represents sequence similarity levels.

Our investigation identified viral RNA from 11 different viruses, most of which possess complete coding regions (Fig. 2). Notably, two of these viral RNAs—Serbia mononega-like virus 1 and Biggievirus Mos11—represent the first documented cases of these viral species in China. The remaining nine viruses, including Culex tritaeniorhynchus rhabdovirus (CTRV), Hubei picorna-like virus 59, Hubei mosquito virus 3, Hubei sobemo-like virus 41, Hubei tombus-like virus 20, the Sichuan mosquito picorna-like virus, Wenzhou sobemo-like virus 4, Zhejiang mosquito virus 3, and Zhee mosquito virus, exhibit close phylogenetic relationships with previously characterized mosquito-associated viruses, though they remain officially unclassified by the ICTV. Comprehensive phylogenetic analysis enabled the classification of these viruses into nine distinct families. Bidirectional clustering analysis of the heatmap and phylogenetic tree revealed three key evolutionary patterns among the viruses: (i) A well-defined cluster comprising Biggievirus Mos11, Hubei picorna-like virus 59, and two sobemo-like viruses (Wenzhou sobemo-like virus 4 and Hubei sobemo-like virus 41), with the sobemo-like pair exhibiting particularly close genetic affinity, indicative of shared ancestry; (ii) A loosely associated group containing Zhee mosquito virus, Sichuan mosquito picorna-like virus, and Zhejiang mosquito virus 3, which displayed measurable but evolutionarily distant relationships; (iii) The unexpected clustering of Hubei mosquito virus 3 with Serbia mononega-like virus 1, potentially attributable to conserved genomic regions, despite the latter’s overall sequence divergence from other viruses. Crucially, all observed clustering patterns remained confined within established viral family boundaries, with no instances of inter-family associations. Host distribution analysis demonstrated that these viruses predominantly infect *Culex* species, including *Culex quinquefasciatus, Culex pipiens pallens,* and *Culex tritaeniorhynchus*. These findings not only underscore the remarkable genetic diversity of mosquito-borne viruses but also highlight their adaptive co-evolution with *Culex* hosts, providing valuable insights into the mechanisms of viral emergence and host specificity (Fig. 3).

#### Botourmiaviridae

**Fig 4:**
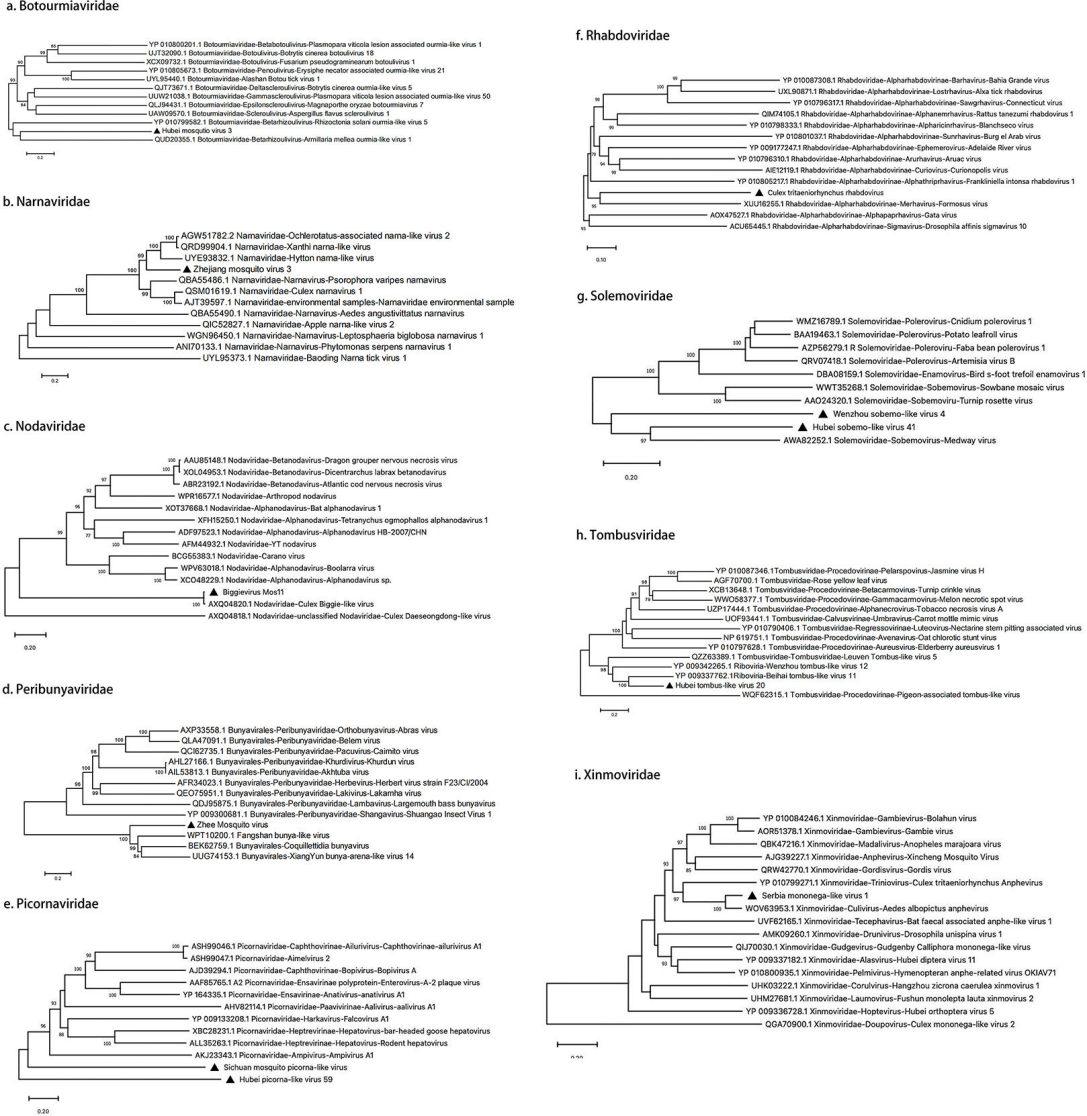
Phylogenetic relationships of nine viral families identified in mosquito samples. Each panel (**a–i**) displays a maximum-likelihood tree constructed from RdRp gene sequences, with sequences obtained in this study indicated by black triangles. The phylogenetic tree was constructed using the NJ method in MEGA 11.1 software, with 1,000 bootstrap repetitions. Branches with bootstrap values exceeding 70% were considered statistically significant.

We discovered a member of the *Botourmiaviridae* family, Hubei mosquito 3 viral RNA. Chinese researchers sequenced this virus in 2016 and submitted the sequence to GenBank (accession KX883460.1), but no detailed taxonomic analysis was carried out. The viral genome comprises a single-stranded positive-sense RNA of 2,011 nt containing two ORFs. ORF1 spans positions 162–1,829 and encodes 555 amino acids; ORF2 spans positions 1,143–1,874 and encodes 243 amino acids. Conserved domain analysis revealed that the two ORFs of *Hubei mosquito virus 3* encode distinct proteins: an RdRp encoded by ORF1 and a hypothetical protein encoded by ORF2. However, the transcriptional and translational mechanisms of these overlapping ORFs remain unclear. The strain 21W-GJD identified in this study shares 99.4% nucleotide sequence identity with strain 3mos6141. The highest amino acid sequence identity (75.5%) was observed with the RdRp of Qianjiang botourmia-like virus 52 within the *Botourmiaviridae* family. We then conducted a comparative analysis of representative viral sequences within the *Botourmiaviridae* family. Phylogenetic analysis based on RdRp sequences showed that Hubei mosquito virus 3 is most closely related to Armillaria mellea ourmia-like virus 1 within the *Betamhizoulivirus* genus, suggesting these viral sequences share a common origin. Therefore, Hubei mosquito virus 3 may represent a novel member of the *Betamhizoulivirus* genus (Fig. 4a).

#### Narnaviridae

We recognized a member of the *Narnaviridae* family, Zhejiang mosquito virus 3 viral RNA. Chinese researchers first discovered this virus in 2016 (12), and subsequently, Gytis Dudas performed detailed genomic functional studies (13). The genome of Zhejiang mosquito virus 3 comprises a single-stranded positive-sense RNA of 3,226 nt containing ORFs: positions 42–3,206 encoding 1,054 amino acids, and positions 54–3,224 encoding 1,056 amino acids. Conserved domain analysis revealed that the ORFs of Zhejiang mosquito virus 3 likely encode two proteins: an RdRp protein (amino acids 1,063–1,827) and a PksD protein (amino acids 664–2,334). However, the transcriptional and translational mechanisms, regulatory features, and molecular weights of these proteins require further investigation. The strain 21W-GJD identified in this study shares 98.3% nucleotide sequence identity with strain mosZJ35354. Additionally, it shares 75.5% nucleotide sequence identity with Ochlerotatus-associated narna-like virus 2 within the *Narnaviridae* family. We then performed a comparative analysis of representative viral sequences within the *Narnaviridae* family. In the RdRp phylogeny, Zhejiang mosquito virus 3 clusters with *Hyiton narna-like virus*, *Xanthi narna-like virus*, and others, and is most closely related to Psorophora varipes narnavirus, which has well-defined genus classification. Therefore, Zhejiang mosquito virus 3 likely represents a novel member of the *Narnavirus* genus within the *Narnaviridae* family (Fig. 4b).

#### Nodaviridae

We identified a member of the *Nodaviridae* family, Biggievirus Mos11 viral RNA. First discovered in the United States in 2016, its sequence was deposited in NCBI (accession KX924639.1). Since then, it has also been reported in Italy, India, and France. This study represents the first report of this virus from China. Biggievirus Mos11 is a positive-strand RNA virus with a genome of 9,171 nt containing three ORFs. ORF1 spans positions 1–6,869 encoding 2,288 amino acids; ORF2 spans positions 6,906–8,132 encoding 408 amino acids; and ORF3 spans positions 8,256–8,903 encoding 215 amino acids. Conserved domain analysis revealed that Biggievirus Mos11 likely encodes five proteins, with key domains including an RNA helicase (amino acids 4,053–4,862) and RdRp (amino acids 5,880–6,662). The strain 21W-GJD identified in this study exhibits 98.7% nucleotide sequence identity with strain IVRI/2017. Additionally, Biggievirus Mos11 shares high-sequence identity with Culex Biggie-like virus within the *Nodaviridae* family. We therefore conducted phylogenetic analysis of representative viral sequences within the *Nodaviridae* family. Phylogenetic analysis based on RdRp sequences showed that Biggievirus Mos11 and Culex Biggie-like virus form an independent clade, exhibiting significant genetic distance from members of the *Alphanodavirus* and *Betanodavirus* genera. This suggests these viruses may represent a novel evolutionary lineage within the *Nodaviridae* family, sharing similar taxonomic status with unclassified members such as Culex Daeseongdong-like virus (Fig. 4c).

#### Peribunyaviridae

We detected a virus from the *Peribunyaviridae* family, specifically the Zhee mosquito viral RNA. Although its genome sequence was submitted to GenBank in 2016 (accession KM817705.1), comprehensive phylogenetic and taxonomic analyses have yet to be conducted. The full-length L segment of this virus is 7,604 nt and contains one *ORF* spanning positions 77-7,408, encoding 2,443 amino acids. Conserved domain analysis revealed that the L segment ORF of Zhee mosquito virus likely encodes a single protein, specifically an RdRp protein spanning amino acid positions 1,994–4,141. The L segment of the Zhee mosquito virus strain 21W-ZC identified in this study shares 98.8% sequence identity with strain XC-1-8. We then conducted a comparative analysis of representative viral sequences within the *Peribunyaviridae* family. In the RdRp phylogeny, Zhee mosquito virus forms a monophyletic group with Fangshan bunya-like virus and Coquillettidia bunyavirus and is most closely related to Shuangao Insect Virus 1 within the *Shangavirus* genus. Therefore, Zhee mosquito virus likely represents a novel member of the *Shangavirus* genus within the *Peribunyaviridae* family (Fig. 4d).

#### Picornaviridae

We identified two members of the *Picornaviridae* family: Hubei picorna-like virus 59 viral RNA and Sichuan mosquito picorna-like viral RNA. The sequence of Hubei picorna-like virus 59 was first submitted to GenBank by Chinese researchers in 2018 (accession NC_033019.1), but no detailed taxonomic analysis was performed. Its genome comprises a single-stranded positive-sense RNA of 9,035 nt containing one *ORF* spanning positions 118–8,832, encoding 2,904 amino acids. Conserved domain analysis revealed that the ORF of Hubei picorna-like virus 59 likely encodes three proteins: an RNA helicase (amino acids 1,237–1,545), an RdRp protein (amino acids 4,945–5,808), and a double-stranded RNA-binding protein (amino acids 6,040–6,222). Additionally, the genomic sequence of Sichuan mosquito picorna-like virus was deposited in GenBank in 2022 (accession MZ556273.1). The viral genome is 10,864 nt and contains three *ORFs* spanning positions 303–2,526, 2,385–3,524, and 3,517–10,773. Conserved domain analysis revealed that the *ORFs* of Sichuan mosquito picorna-like virus likely encode two proteins: an RNA helicase spanning amino acids 2,015–2,392 and an RdRp spanning amino acids 4,655–5,623. The Hubei picorna-like virus 59 strain 21W-ZC identified in this study shares 95.3% nucleotide sequence identity with strain spider133744. Meanwhile, the Sichuan mosquito picorna-like virus strain 21W-ZC shares 99.8% sequence identity with strain mos091. We then conducted a comparative analysis of representative viral sequences within the *Picornaviridae* family. In the RdRp phylogeny, Hubei picorna-like virus 59 and Sichuan mosquito picorna-like virus, although belonging to different clades, are most closely related to Ampivirus A1 within the *Ampivirus* genus, indicating these viral sequences share a common origin. Therefore, these viruses may represent novel members of the *Ampivirus* genus within the *Picornaviridae* family (Fig. 4e).

#### Rhabdoviridae

We detected a virus from the *Rhabdoviridae* family, CTRV viral RNA, which was first identified in Japan, and its sequence information was submitted to NCBI in 2014 (accession AB604791.1). This virus has since been reported from China. The viral genome comprises a single-stranded negative-sense RNA of 11,190 nt containing five ORFs: nucleotide positions 98–1,516, 1,669–2,463, 2,508–3,029, 3,101–4,612, and 4,682–11,129, encoding nucleoprotein, phosphoprotein, matrix protein, glycoprotein, and large protein, respectively. The strain 21W-ZC identified in this study exhibits 99.4% nucleotide sequence identity with strain YX204. A comparative analysis of representative viral sequences within the *Rhabdoviridae* family was performed. Phylogenetic analysis based on RdRp sequences revealed that CTRV is most closely related to Formosus within the *Merhavirus* genus of the *Alpharhabdovirinae* subfamily. Therefore, CTRV represents a member of the *Merhavirus* genus (Fig. 4f).

#### Solemoviridae

We detected two viruses from the *Solemoviridae* family: Hubei sobemo-like virus 41 viral RNA and Wenzhou sobemo-like virus 4 viral RNA. Their sequences were initially determined and submitted to GenBank by Zhang et al. in 2017 (accessions KX882766.1, KX882831.1). The Hubei sobemo-like virus 41 genome comprises a single-stranded positive-sense RNA of 2,787 nt containing two ORFs. ORF1 spans nucleotide positions 64–1,497 encoding 477 amino acids, while ORF2 spans positions 1,575–2,693 encoding 272 amino acids. Conserved domain analysis revealed that ORF2 of Hubei sobemo-like virus 41 encodes an RdRp protein spanning amino acid positions 1,797–2,465. The Wenzhou sobemo-like virus 4 genome comprises 2,961 nt containing two ORFs. ORF1 spans nucleotide positions 68–1,837 encoding 589 amino acids, while ORF2 spans positions 1,579–2,904 encoding 441 amino acids. Conserved domain analysis revealed that ORF2 of Wenzhou sobemo-like virus 4 encodes an RdRp protein spanning amino acid positions 1,627–2,736. The Hubei sobemo-like virus 41 strain 21W-GJD identified in this study exhibits 98.3% nucleotide sequence identity with strain 3mos6151. Wenzhou sobemo-like virus 4 strain 21W-ZC exhibits 99.9% nucleotide sequence identity with strain 18LZ-22, while showing <93% identity with strains from other regions. A comparative analysis of representative viral sequences within the *Solemoviridae* family was performed. Phylogenetic analysis based on RdRp sequences revealed that Hubei sobemo-like virus 41 is most closely related to Medway virus within the *Sobemovirus* genus and forms a monophyletic group with Wenzhou sobemo-like virus 4. Therefore, both viruses likely represent novel members of the *Sobemovirus* genus (Fig. 4g).

#### Tombusviridae

Hubei tombus-like virus 20 is a member of the *Tombusviridae* family, originally discovered in spiders. The complete genome sequence was deposited in GenBank in 2017 (accession KX883163.1). The viral genome comprises a single-stranded positive-sense RNA of 4,832 nt containing four ORFs. Conserved domain analysis revealed: ORF1 (nucleotide positions 19–1,173) encoding a hypothetical protein of 384 amino acids, ORF2 (positions 849–1,859) encoding a hypothetical protein of 336 amino acids, ORF3 (positions 1,676–2,977) encoding an RdRp protein of 433 amino acids, and ORF4 (positions 2,982–4,718) encoding a hypothetical protein of 578 amino acids. However, the transcriptional and translational mechanisms of these proteins require further investigation. The strain 21W-GJD identified in this study exhibits 95.3% nucleotide sequence identity with spider131362. A comparative analysis of representative viral sequences within the *Tombusviridae* family was performed. Phylogenetic analysis based on RdRp sequences revealed that Hubei tombus-like virus 20 is most closely related to Beihai tombus-like virus 11 and Wenzhou tombus-like virus 12, forming a monophyletic group that clusters with the unclassified *Tombusviridae* member Leuven Tombus-like virus 5. Therefore, Hubei tombus-like virus 20 likely represents a novel member of the *Tombusviridae* family (Fig. 4h).

#### Xinmoviridae

We discovered a viral RNA belonging to the *Xinmoviridae* family, named Serbia mononega-like virus 1. It was first identified in Serbia in 2020, and its sequence information has been submitted to the NCBI database (accession number MT822181.1). This study represents the first report of this virus from China. The Serbia mononega-like virus 1 genome comprises a single-stranded negative-sense RNA of 12,933 nt containing four ORFs: nucleotide positions 386–1,693, 2,207–4,213, 4,262–6,178, and 6,417–12,578, encoding nucleoprotein, hypothetical protein, glycoprotein, and RdRp, respectively. The strain 21W-ZC identified in this study exhibits 90% sequence identity and 89.4% nucleotide sequence identity with known strains. A comparative analysis of representative viruses from all genera within the *Xinmoviridae* family was performed. Phylogenetic analysis based on polyprotein sequences revealed that Serbia mononega-like virus 1 forms a monophyletic group with Aedes albopictus anphevirus within the *Culivirus* genus. Therefore, this virus likely represents a novel member of the *Xinmoviridae* family and *Culivirus* genus (Fig. 4i).

### Viral coexistence across mosquito species

**Fig 5:**
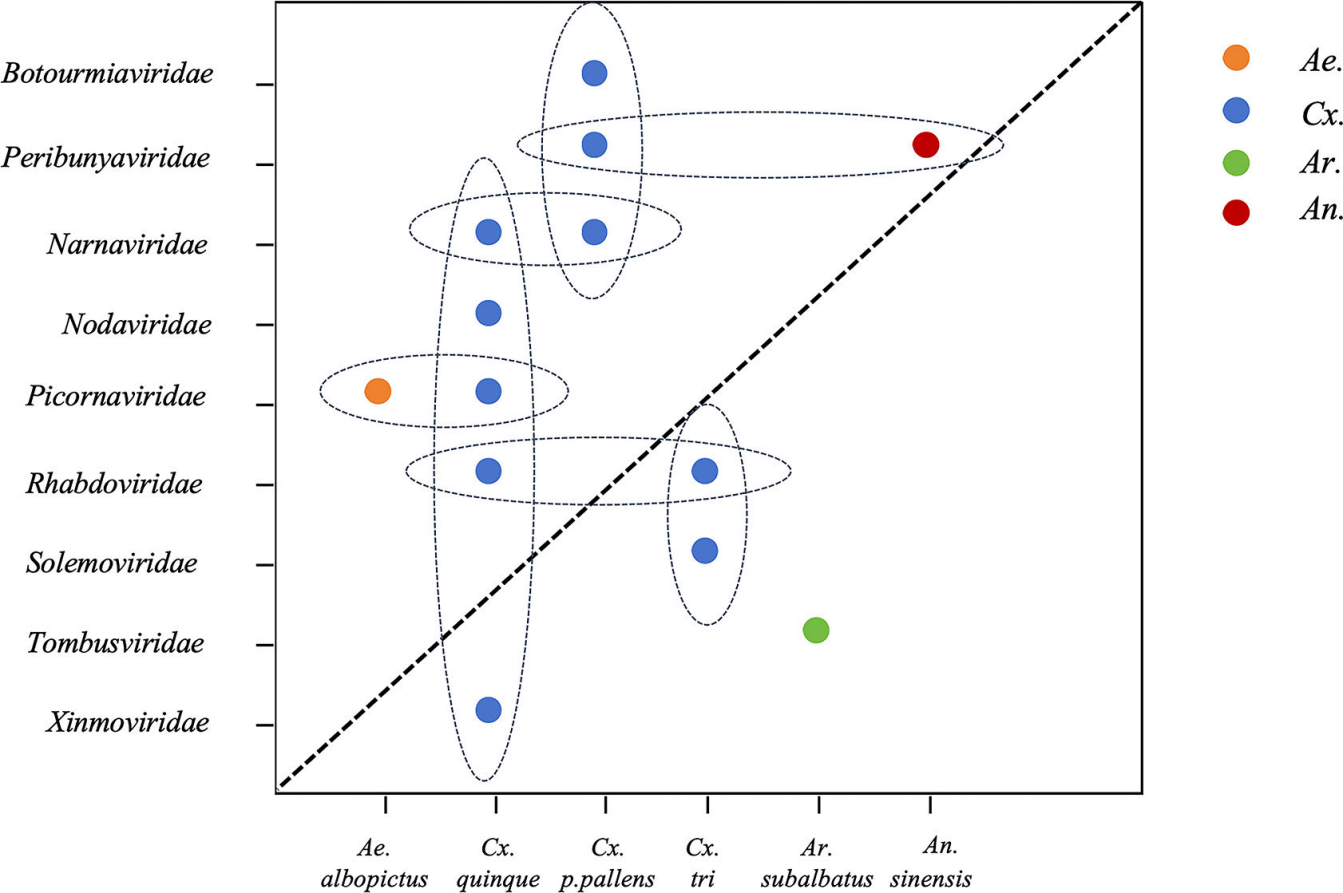
The figure presents the ecological relationships between virus families (y-axis) and their mosquito vectors (x-axis). Virus-mosquito associations are indicated by colored data points, with dashed ellipses highlighting significant vector-virus groupings observed in our study.

The number of viral species varied among mosquito species. *Culex quinquefasciatus* harbored the highest viral diversity with five species, while *Culex pipiens pallens*, *Culex tritaeniorhynchus*, *Armigeres subalbatus*, *Anopheles sinensis*, and *Aedes togoi* harbored three, three, one, one, and one viral species, respectively (Fig. 5).

In the six *Culex quinquefasciatus* libraries (L8-L13), viral reads predominantly originated from four families: *Picornaviridae* (30.1%), *Narnaviridae* (27.4%), *Xinmoviridae* (15.6%), and *Nodaviridae* (13.5%). Species-level analysis revealed that these reads primarily comprised Hubei picorna-like virus 59, Zhejiang mosquito virus 3, Serbia mononega-like virus 1, Biggievirus Mos11, and CTRV.

In the seven *Culex pipiens pallens* libraries (L1–L7), three viruses were identified: Zhee mosquito virus, Hubei mosquito virus 3, and Zhejiang mosquito virus 3. Three viruses were detected in the *Culex tritaeniorhynchus* libraries (L16 and L17): Hubei sobemo-like virus 41, Wenzhou sobemo-like virus 4, and CTRV. Hubei tombus-like virus 20 was detected in *Armigeres subalbatus* libraries (L14 and L15), Zhee mosquito virus in the *Anopheles sinensis* library (L18), and Sichuan mosquito picorna-like virus in the *Aedes togoi* library (L19).

Several viral species exhibited cross-species detection across different mosquito hosts. Specifically, Zhee mosquito virus was detected in both *Culex pipiens pallens* and *Anopheles sinensis*, *Zhejiang mosquito virus 3* in both *Culex quinquefasciatus* and *Culex pipiens pallens*, and CTRV in both *Culex quinquefasciatus* and *Culex tritaeniorhynchus*.

### Viral ecology analysis

Host composition analysis revealed that the *Culex* genus (*Culex pipiens*, *Culex quinquefasciatus*, and *Culex tritaeniorhynchus*) comprised 89.8% (6,568/7,311) of the total sample, with *Culex pipiens pallens* (3,050 individuals) and *Culex quinquefasciatus* (2,862 individuals) as dominant species. *Culex mosquitoes* carried nine viral species, whereas each of the three other taxa examined—*Aedes*, *Anopheles*, and *Armigeres*—harbored only one. The Mann-Whitney U test revealed no statistically significant difference in viral species distribution at the species level (U = 8.5, *P* = 0.157). Permutation testing at the individual level similarly revealed no significant differences (*P* = 0.089). The Simpson diversity index indicated higher viral community diversity in the *Culex* genus (1-D = 0.832 vs. 0.507). This pattern may reflect the broad host range of *Culex* mosquitoes (feeding on birds, livestock, and humans) and high population density (comprising 89.8% of the sample). As anthropophilic vectors, *Culex* mosquito larvae preferentially develop in organically enriched aquatic habitats, potentially facilitating multi-host viral transmission. Notably, although *Aedes togoi* harbored only one viral species (Sichuan mosquito picorna-like virus), its small sample size (128 specimens) yielded a detection rate of 7.8/1,000—substantially higher than the 1.4/1,000 observed in *Culex*. This disparity likely stems from our nighttime, livestock-shelter trapping protocol, which favors endophilic species but under-samples day-active or coastal vectors such as *Aedes togoi*. The resulting sample-size imbalance precludes reliable estimation of viral prevalence in this mosquito.

**Fig 6:**
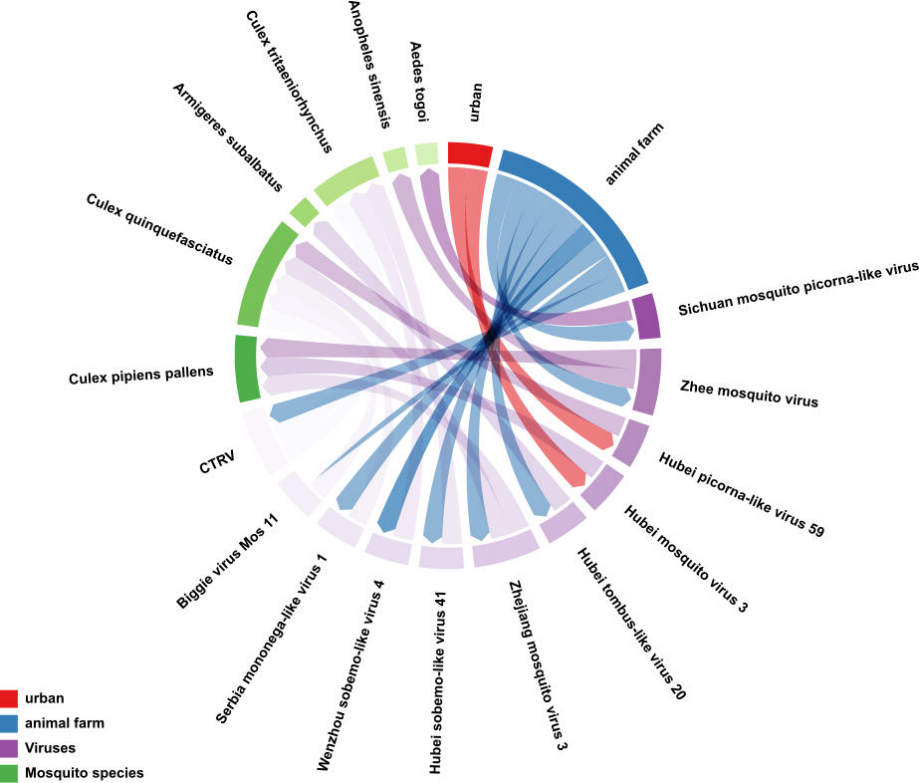
Host-virus-environment transmission network chord diagram. The chord diagram visualizes transmission networks among host, virus, and environment components. Environmental sampling sites are color-coded (red: urban; blue: animal farms), viral species are represented in purple gradients, and mosquito vectors are shown in green variants, demonstrating their ecological associations.

Viral diversity analysis revealed significantly higher viral prevalence in agricultural areas compared to urban areas (permutation test: *P* < 0.001). Average viral species density reached 5.02 per thousand individuals in agricultural areas, significantly exceeding 2.94 per thousand in urban areas. PERMANOVA analysis confirmed significant differences in viral community structure between agricultural and urban sites (F = 15.7, *P* = 0.010). Agricultural sites harbored multiple cross-species viruses (e.g., Zhee mosquito virus and CTRV, both detected across two *Culex* species). This elevated viral diversity likely reflects multiple ecological factors: livestock operations facilitate cross-species viral transmission, organic-rich wastewater systems enhance viral persistence, and high-density host populations increase vector-host contact rates. Conversely, reduced host diversity and urban pollutants may limit viral establishment in urban environments (Fig. 6).

### Structural analysis of novel virus-encoded proteins

**Fig 7:**
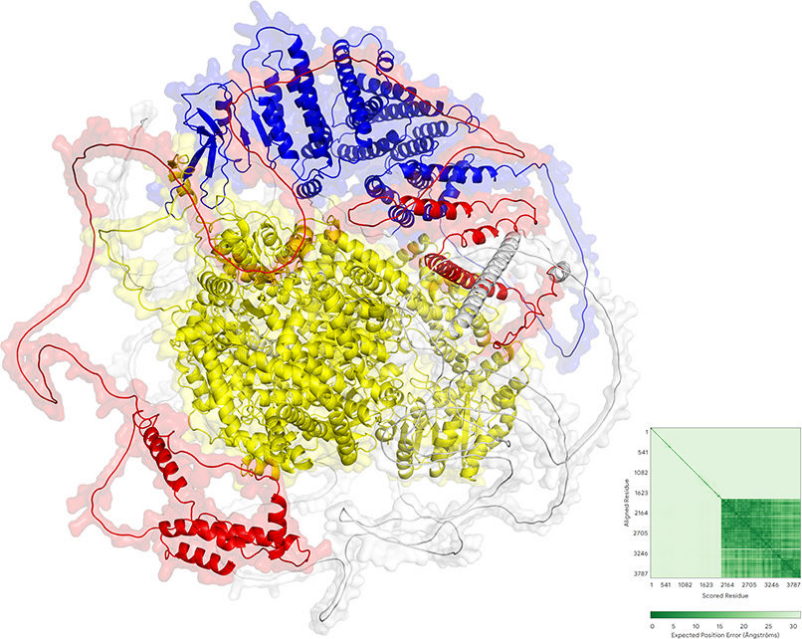
The three-dimensional structure of Serbia mononega-like virus 1 is presented, with distinct functional domains color-coded as follows: RdRp (yellow), nucleoprotein (red), and glycoprotein (blue). The accompanying PAE plot (right panel) displays residue-residue correlations using a green color gradient, with darker green indicating higher confidence in structural predictions. The spatial coordinates demonstrate either protein-protein interactions or structural mapping relationships.

Structural and functional analyses of Serbia mononega-like virus 1 protein-coding regions were performed using AlphaFold predictions. The virus exhibits a complex multi-domain architecture (Fig. 7). The RdRp domain displays a characteristic compact globular topology typical of viral polymerases, with the highly conserved GDD catalytic motif positioned at the structural core for optimal catalytic geometry. The N-terminal and C-terminal regions exhibit distinct folding patterns, suggesting roles in genome replication regulation or host factor interactions. The nucleoprotein domain adopts a compact conformation, suggesting involvement in genome packaging or host factor recruitment. The glycoprotein domain displays unique folding patterns that may facilitate host cell recognition and viral entry. Predicted alignment error (PAE) analysis revealed extremely high model confidence (PAE <10 Å) in the core catalytic region, particularly within the RdRp domain. Analysis revealed extensive interdomain interactions, particularly in the C-terminal region, suggesting potential allosteric regulation of enzyme activity. The N-terminal region exhibited higher predicted errors, suggesting structural flexibility that may be crucial for functional regulation.

**Fig 8:**
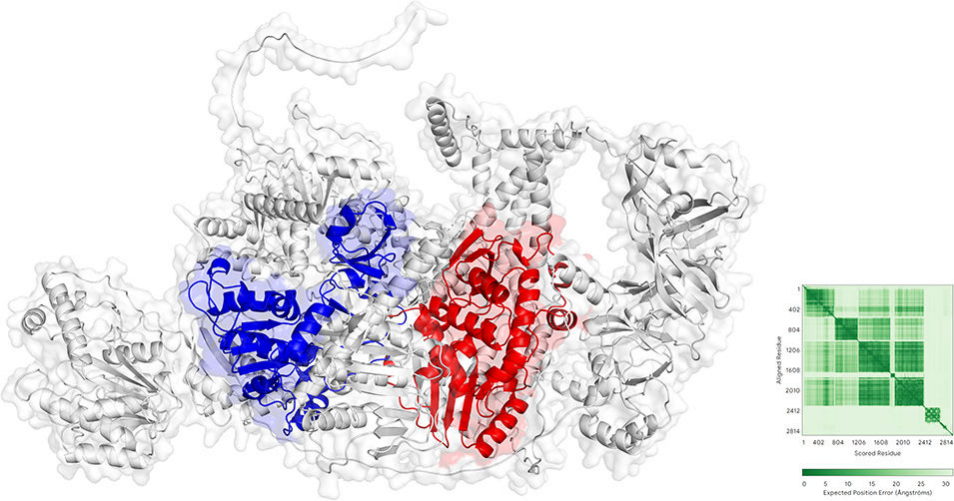
The predicted structure of Biggievirus Mos11 displays key functional domains: RNA helicase (blue) and RNA-dependent RNA polymerase (RdRp, red). The accompanying alignment error plot (right) indicates prediction confidence through a green color gradient, with darker hues representing higher reliability in residue-residue correlations. Structural coordinates reveal potential inter-domain interactions or spatial mapping relationships.

Two major functional domains were identified in Biggievirus Mos11 (Fig. 8). The RNA helicase domain contains a typical ATP-binding site and multiple conserved helicase motifs, likely facilitating viral genome replication through ATP-dependent nucleic acid unwinding. This activity is essential for viral RNA processing during infection. The RdRp domain contains classic GDD and SDD catalytic motifs, characteristic of RNA-dependent RNA polymerase activity. PAE analysis revealed confidence distribution across the protein structure. Both RNA helicase and RdRp domains exhibit excellent internal structural stability (PAE <10 Å) with distinct interdomain interfaces, suggesting functional complex formation. However, while the helicase domain shows accurate internal conformation predictions, it exhibits moderate PAE values with other domains, suggesting participation in viral regulation through dynamic conformational changes.

### Nucleic acid detection of novel viruses in mosquito samples

Nucleic acid detection was performed for two novel viruses across all 165 mosquito sample pools. qRT-PCR analysis revealed 8 and 16 positive pools for Serbia mononega-like virus 1 and Biggievirus Mos11, respectively. Both viruses were detected exclusively in *Culex quinquefasciatus*. MIRs were 0.34% for Serbia mononega-like virus 1 and 0.68% for Biggievirus Mos11 (Table 2).

**TABLE 2:** qRT-PCR to determine the two newly identified viruses in mosquito samples of Yantai, Shandong Province, in 2021

Species	No. of mosquitoes	Site	No. of pools	Library	Serbia mononega-like virus 1		Biggievirus Mos11	
					No. of positive pools	MIR %	No. of positive pools	MIR %
*Culex pipiens pallens*	486	Urban area	10	L1	0	0	0	0
	2,564	Animal farm	51	L2- L7	0	0	0	
*Culex quinquefasciatus*	502	Urban area	10	L8	0	0	0	0
	2,360	Animal farm	48	L9- L13	8	0.34	16	0.68
*Armigeres subalbatus*	944	Animal farm	19	L14 L15	0	0	0	0
*Culex tritaeniorhynchus*	656	Animal farm	14	L16 L17	0	0	0	0
*Anopheles sinensis*	471	Animal farm	10	L18	0	0	0	0
*Aedes albopictus*	128	Animal farm	3	L19	0	0	0	0
Total	8111		165	19	8	0.1	16	0.2

## DISCUSSION

Mosquito-borne viruses represent a persistent global public health threat, with outbreaks causing a substantial burden of disease in regions, healthcare system strain, and causing socioeconomic losses (14). Climate change has expanded suitable mosquito habitats, while increased international travel and trade facilitate cross-border pathogen dispersal. Rapid urbanization and population densification have enhanced the efficiency of viral transmission, escalating global mosquito-borne virus transmission risk and triggering multiple regional outbreaks (15). Historical examples include the 20th-century DENV outbreak in Southeast Asia, causing over one million severe cases, the transcontinental spread of WNV in North America, and the 2016 ZIKV-associated microcephaly outbreak, all demonstrating the potential for mosquito-borne pathogens to become international public health emergencies (16, 17). Current surveillance data indicate that natural mosquito viral diversity far exceeds our understanding. Metagenomic sequencing has identified over 500 novel mosquito-associated viruses, some with cross-species transmission potential (18). This approach broadens the temporal and spatial scope of pathogen surveillance while providing molecular foundations for targeted control strategies through viral evolutionary analysis (19, 20).

This study systematically identified and analyzed viral genomes from six common mosquito species in Yantai City, Shandong Province, China. High-throughput sequencing yielded complete coding sequences or RdRp gene sequences for 11 viruses, including two viruses newly identified in China. These viruses span nine viral families, confirming extensive viral diversity in mosquito vectors. These findings align with global studies. For example, Williams et al. identified six viruses from four families in three mosquito species from northern Australia (21), while Schilling et al. identified over 10 viruses from five families in two mosquito species from Greenland (22). These studies collectively demonstrate that mosquitoes serve as important viral reservoirs, participating in viral replication and transmission cycles. Viral composition can vary significantly among the same mosquito species within geographical regions. Wang et al. (2021) revealed that eight viral families in four common Shandong mosquito species were almost entirely distinct from those identified here (23). This underscores the complexity of mosquito viral communities, which exceeds previous understanding. Most viruses identified here are likely mosquito-specific and pose no direct threat to human or animal health. This aligns with known mosquito-borne virus ecology, as most medically important viruses replicate at low levels in mosquitoes and proliferate only under specific epidemiological conditions. Multiple studies have shown that known human and animal pathogens make up only a small fraction of mosquito viral genomes. One of the viral RNAs detected in our study, Hubei tombus-like virus 20, is likely a plant virus. Given that the Tombusviridae family is predominantly associated with plants, it is highly probable that this virus primarily infects plant hosts rather than mosquitoes. The detection of Hubei tombus-like virus 20 RNA in our mosquito samples is more likely a result of the mosquitoes ingesting plant material containing the virus, rather than the virus replicating within the mosquitoes themselves. This scenario is consistent with the feeding behavior of mosquitoes, which can ingest plant sap or nectar as part of their diet. Therefore, the presence of Hubei tombus-like virus 20 RNA in our samples should be interpreted as the detection of viral RNA fragments originating from plant sources, rather than evidence of active viral replication or infection within the mosquito hosts. While the majority of detected viral RNAs may not pose direct threats to humans or animals, some identified viruses warrant further investigation due to their potential pathogenicity. In addition, Zhee mosquito virus (*Peribunyaviridae*) may have pathogenic potential, as phylogenetic analysis reveals close relationships to *Shangavirus* genus members. *Shangavirus* and *Orthobunyavirus* (e.g., La Crosse virus and Oropouche virus) share similar genomic structures, with the latter causing human fever and encephalitis (24, 25). The *Merhavirus* member CTRV detected here has been found only in mosquito hosts with no reported vertebrate infections. However, phylogenetic analysis reveals that CTRV is closely related to pathogenic genera within the *Alpharhabdovirinae* subfamily, including Chandipura virus (*Vesiculovirus*) and bovine ephemeral fever virus (*Ephemerovirus*) (26, 27). This suggests CTRV may possess cross-species transmission risk or pathogenic evolutionary potential. The pathogenic potential of these viruses requires experimental verification.

High-throughput metagenomic sequencing enabled assembly of complete coding sequences for 11 known viruses from mosquito samples. Although all viruses are recorded in public databases, existing sequences predominantly derive from recent publications with limited reference genomic information. Systematic genomic analysis revealed significant differences in nucleic acid type: three viruses (Zhee mosquito virus, CTRV, and Serbia mononega-like virus 1) are single-stranded negative-sense RNA viruses, while eight are single-stranded positive-sense RNA viruses. This positive-sense RNA virus predominance may reflect mosquitoes’ ecological role as hosts for plant- and insect-specific viruses. Similar patterns have been reported in comparable international studies (28). Regarding viral classification, CTRV and Serbia mononega-like virus 1 have established genus-level classifications, while nine viruses lack systematic taxonomic positioning. All viruses encode complete RdRp domains. Given RdRp’s high conservation and sequence length advantages, phylogenetic analysis based on RdRp sequences successfully classified these viruses into genus-level taxonomic units. This analysis refines viral taxonomic status while providing insights for future biological studies. This study identified molecular evidence of cross-species transmission. Three viruses (Hubei picorna-like virus 59, Hubei tombus-like virus 20, and Hubei sobemo-like virus 41) were previously reported from arachnid hosts. However, highly homologous sequences were detected in *Culex quinquefasciatus*, *Armigeres subalbatus*, and *Culex tritaeniorhynchus*. This suggests that these viruses may breach host barriers or participate in complex transmission networks within natural ecosystems. However, food chain transmission (e.g., mosquitoes preying on spiders) cannot be excluded based on current data.

This study reports the first identification of two viral RNAs in China: Serbia mononega-like virus 1 and Biggievirus Mos11. Serbia mononega-like virus 1 was previously reported only from Serbia, with this study expanding its geographical distribution to China. Genomic analysis revealed 96.4% and 95.0% sequence identity for glycoprotein and RdRp between strains, respectively, indicating conserved protein structures despite regional differences. This virus belongs to the *Culivirus* genus (*Xinmoviridae*) and may exhibit mosquito-borne transmission, although mammalian infection or human pathogenicity remains unclear. Biggievirus Mos11 has been reported from North America, Western Europe, and South Asia, with this study confirming its first detection in East Asia. RNA helicase and RdRp sequences exhibit >99.1% identity with known strains, indicating high evolutionary conservation. Phylogenetic analysis suggests membership in the *Alphanodavirus* genus (*Nodaviridae*), whose members primarily infect insects but not vertebrates. Local mosquito population testing revealed positive rates of 0.34% and 0.68% for both viruses, respectively, indicating local adaptation and stable regional circulation. This aligns with previous findings for Hubei mosquito virus 2 (HMV2) in Shandong Province (29). The viral RNAs of the two novel viruses (Serbia mononega-like virus 1 and Biggievirus Mos11) were detected exclusively in *Culex quinquefasciatus* in this study. (This finding is specific to these viruses and does not imply that all viruses discussed in this manuscript were detected in *Culex quinquefasciatus*), suggesting potential cross-species infectivity. This expands understanding of viral host range, with potential public health implications warranting further investigation.

Mosquitoes serve as important arbovirus vectors and harbor complex viral communities. Multiple viruses were detected across mosquito species: *Culex quinquefasciatus* harbored five viruses from different families, while *Culex pipiens pallens* and *Culex tritaeniorhynchus* each harbored three viruses. This viral diversity may reflect mosquito feeding behavior. Female mosquitoes, in addition to feeding on plant juices for energy and nutrients, require blood meals for reproduction, which can expose them to a broader range of potential viral sources. Male mosquitoes, on the other hand, primarily obtain their carbohydrates from plant juices, including nectar and other plant fluids, which provide the necessary energy for their daily activities and survival (30, 31). This broad feeding range may facilitate exposure to and acquisition of multiple viruses. While pooled sample detection determines species-level viral profiles, it cannot distinguish simultaneous multi-viral infections in individual mosquitoes. Certain viruses (Zhee mosquito virus and Zhejiang mosquito virus 3) were detected across multiple mosquito species, suggesting broad vector specificity, cross-species transmission potential, and expanded vector ranges. These findings suggest strong environmental adaptability and enhanced transmission capabilities for these viruses. While our study detected viral RNA in several mosquito species, it is notable that some viruses were not detected in others. This could be due to differences in ecological niches and behaviors among mosquito species. For instance, certain mosquito species may have feeding preferences or habitats that reduce their exposure to viral sources. Additionally, variations in vector competence and susceptibility to viral infection could also play a role. Future studies should investigate these factors in more detail to elucidate the reasons behind the observed distribution patterns of viral detection among different mosquito species.

This study has several limitations. First, nighttime collection may have overlooked diurnal species such as *Aedes albopictus*, potentially resulting in incomplete viral diversity data. Second, without viral enrichment and isolation, sequences may reflect only high-abundance viral populations, precluding comprehensive assessment of low-abundance virus infectivity and pathogenicity. Third, reliance solely on metagenomic sequencing without viral isolation, cultivation, or animal infection experiments precludes confirmation of viral infectivity and pathogenic potential. While our study detected viral RNA from 11 different viruses, it is important to note that the presence of viral RNA does not definitively confirm the presence of infectious virus particles. For example, previous studies have reported the detection of viral RNA from arboviruses such as Zika, dengue, and CHIKVs in mosquitoes, even when no infectious virus was present (e.g., mosquitoes exposed to inactivated virus). This highlights the need for additional studies to confirm the presence of infectious viruses, including attempts to isolate live virus particles and conduct further experiments to assess their infectivity and pathogenic potential.

### Conclusion

This study reveals complex mosquito-borne viral diversity in Yantai, Shandong Province, with potential public health significance. Metagenomic analysis revealed widespread distribution of multiple mosquito-borne viruses within the *Culex* genus, with some exhibiting cross-host transmission characteristics that reflect virus-vector co-evolution. Certain viruses share phylogenetic relationships with known pathogens, indicating the need for cross-species transmission monitoring. These findings enhance understanding of mosquito-borne viral ecology while providing evidence for regional vector-borne disease control.

### Highlights:

- This study uses metagenomics to systematically analyze viral communities in mosquitoes from Yantai, Shandong, identifying 11 viral species across 9 families, with *Serbia mononega-like virus 1* and *Biggievirus Mos11* being first reported in China.
- Phylogenetic analysis based on RdRp sequences clarifies the taxonomic status of the identified viruses, with several potentially representing novel genera or species, enhancing understanding of their evolutionary 8 relationships.
- Host distribution analysis shows *Culex quinquefasciatus* harbors the 10 greatest viral diversity, and some viruses exhibit cross-species transmission potential, indicating host specificity and transmission complexity.
- Viral ecology analysis reveals significantly higher viral diversity in agricultural areas than urban areas, and qRT-PCR confirms the prevalence of the two novel viruses in local *Culex quinquefasciatus* populations with minimum infection rates of 0.28% and 0.56% respectively.

## Data Availability

The 11 viral genome sequences obtained in this study (PV890469-71, PV849722, PV948199-200, PV920663-64, PQ753144, and PQ767083/86) have been submitted to the GenBank database for permanent storage.
